# Early versus late administration of long-acting injectable antipsychotic agents among patients with newly diagnosed schizophrenia: an analysis of a commercial claims database

**DOI:** 10.1097/YIC.0000000000000452

**Published:** 2023-02-08

**Authors:** John M. Kane, Anna Chen, Sangtaeck Lim, Marko A. Mychaskiw, Marc Tian, Yitong Wang, Mark Suett, Jose M. Rubio

**Affiliations:** a The Zucker Hillside Hospital, Northwell Health, Glen Oaks; b Donald and Barbara Zucker School of Medicine at Hofstra/Northwell, Hempstead; c Feinstein Institutes for Medical Research, Institute of Behavioral Science, Manhasset, New York; d Teva Branded Pharmaceutical Products R&D, Inc., Global Health Economics and Outcomes Research; e Teva Branded Pharmaceutical Products R&D, Inc., Clinical and Real World Evidence Statistics, West Chester, Pennsylvania, USA; f Teva UK Limited, Global Medical Affairs, Harlow, UK

**Keywords:** healthcare costs, healthcare resource utilization, insurance claim review, long-acting injectable antipsychotic agents, newly diagnosed schizophrenia, time factors

## Abstract

This study was designed to assess healthcare resource utilization (HCRU) and costs in patients with newly diagnosed schizophrenia based on timing and context of long-acting injectable antipsychotic agent (LAI) initiation. Using claims data, patients (aged 18–40 years) with first schizophrenia diagnosis January 2013–September 2019 (index date), no LAI or oral antipsychotic agent claims during 12-month preindex period, and continuous benefit enrollment from 12 months before index date to 12 months after first LAI administration were identified. Patients were grouped based on timing [early (≤1 year after index date) vs. late] and circumstances [reactive (after schizophrenia-related event) vs. proactive] of LAI initiation. Of 1290 patients with at least one LAI claim, 306 met criteria for early (*n* = 204; reactive, *n* = 107; proactive, *n* = 97) and late (*n* = 102; *n* = 75; *n* = 27) initiation. HCRU and costs were numerically lower in early versus late groups, and significantly lower for proactive initiation in both groups. Comparing worst-case (late-reactive) and best-case (early-proactive) scenarios, the average annual cost difference was $7195.13 (*P* = 0.0233), with major drivers being emergency department ($171.28; *P* < 0.05) and other outpatient ($2845.73; *P* < 0.00001) visits. In addition to the clinical advantages previously described in the literature, the proactive use of LAIs in early-phase schizophrenia is associated with lower healthcare costs.

## Introduction

Schizophrenia is a complex, chronic psychiatric disorder characterized by impaired perception, cognitive ability, emotional expression, and social interactions (Patel *et al.*, 2014; Kahn *et al.*, 2015). Common symptoms of schizophrenia include delusions, hallucinations, and impaired communication and executive function (Patel *et al.*, 2014; Kahn *et al.*, 2015). Schizophrenia affects 0.6–1.9% of the USA population is associated with substantial burden for patients and their families, and is ranked among the top 20 illnesses that contribute to the total global burden of disease (Patel *et al.*, 2014; GBD 2017 Disease and Injury Incidence and Prevalence Collaborators, 2018).

Antipsychotic treatment, such as oral antipsychotic agents (OAs) and newer long-acting injectable antipsychotic agents (LAIs), is the mainstay of therapy (Patel *et al.*, 2014). Regarding relapse prevention and control of psychotic symptoms, LAIs confer several advantages over OAs (Subotnik *et al.*, 2015; Kishimoto *et al.*, 2021). Relapse prevention in schizophrenia is of the utmost importance because attenuated responses to schizophrenia treatment and treatment resistance have been observed following multiple relapses (Takeuchi *et al.*, 2019; Taipale *et al.*, 2022). In addition, LAIs have demonstrated improved patient outcomes, including reduced medication nonadherence, and reduced healthcare utilizations and related costs (Kishimoto *et al.*, 2013, 2021; Munday *et al.*, 2019). Treatment nonadherence is most common early in the course of illness (Tiihonen *et al.*, 2011; Rubio *et al.*, 2021). It is during this phase of the illness when preventing relapses is most crucial, to avoid potential accumulation of morbidity later. Furthermore, LAI utilization in early-phase schizophrenia provides a clinically meaningful reduction in time to first hospitalization, an economically important outcome (Tiihonen *et al.*, 2011; Kane *et al.*, 2020).

Given the clear advantage of LAIs in preventing relapses, and the high risk and impact of relapse during the early phase of the illness, LAIs are increasingly seen as an appropriate treatment option for early-phase schizophrenia, instead of only in patients who have poor adherence (Schreiner *et al.*, 2015; Sajatovic *et al.*, 2018; Florida Medicaid Drug Therapy Management Program, 2020; Kane *et al.*, 2020). Furthermore, early use of LAI therapy is associated with neuroprotection that can further improve patient outcomes (Stevens *et al.*, 2016).

Although LAIs have demonstrated improved clinical effectiveness and adherence versus OAs, they are consistently underutilized for the treatment of patients with schizophrenia (Pilon *et al.*, 2017; Greene *et al.*, 2018). It is of particular interest to further investigate the impact of early versus later LAI initiation in the disease course on healthcare utilization and costs among patients with newly diagnosed schizophrenia (Stahl, 2014), as well as in a commercially insured population (Fitch *et al.*, 2014).

Given the possible advantages of initiating LAIs in the early phase of the illness, this US-based, retrospective claims database study was designed to assess whether earlier use of LAIs is associated with advantages in healthcare resource utilization (HCRU), as well as healthcare costs. In addition, this study sought to determine whether LAIs used proactively as relapse prevention tools, versus reactively following a relapse, corresponded to lower schizophrenia-related HCRU and costs among patients. We hypothesized that earlier implementation, as well as proactive initiation of an LAI, would provide the greatest HCRU- and cost-benefits to the patient population.

## Methods

### Study design

Patients with newly diagnosed schizophrenia [International Classification of Diseases (ICD)-9 and ICD-10] were followed using claims data from the US-based IBM MarketScan Commercial and Medicare Supplemental databases (IBM Watson Health, 2020) from 1 January 2012 to 30 September 2020 (Supplementary Fig. 1, Supplemental Digital Content 1, http://links.lww.com/ICP/A112). The IBM MarketScan databases use deidentified patient-level health data to capture information across the continuum of care (IBM Watson Health, 2020). Its Commercial Database consists of medical and pharmacy claims data for individuals enrolled in a variety of fee-for-service, fully capitated, and partially capitated health plans, including preferred provider organizations, exclusive provider organizations, and point-of-service plans (IBM Watson Health, 2020). The IBM MarketScan Medicare Supplemental Database covers medical and pharmacy claims for individuals enrolled in Medicare Supplemental health insurance plans (IBM Watson Health, 2020).

Key inclusion criteria were the first diagnosis of schizophrenia from 1 January 2013 to 30 September 2019 (index date), ≥1 LAI claim after index date, 18–40 years old, no schizophrenia diagnosis and no LAI or OA claims during 12-month preindex period (i.e. newly diagnosed schizophrenia), and continuous enrollment in a medical and pharmacy insurance plan from 12 months before index date through 12 months after first LAI administration (<30-day gaps allowed). The rationale for these criteria was to ensure that included patients were not enrolled in another insurance plan in which they could have received an earlier schizophrenia diagnosis or treatment as well as to ensure that patients remained enrolled after their first LAI administration in order to capture any HCRU and costs accrued after their first LAI administration. Patients were grouped based on the timing of their first LAI administration (early, ≤1 year after first schizophrenia diagnosis/index date; late, >1 year after) and context of LAI initiation [reactive after schizophrenia-related hospitalization (medical claim coded as nonemergency inpatient hospital visit) or emergency department (ED; medical claim coded as emergency hospital visit) visit; proactive, before hospitalization or ED visit]. In addition to schizophrenia-related hospitalizations and ED visits, medical claims were also categorized as office visits (coded as office visit), or other outpatient visits (outpatient medical claims without office visit, hospitalization, or ED coded).

### Outcomes

Outcomes of interest included proportion of patients with newly diagnosed schizophrenia who initiated an LAI early or late; proportion of patients within early and late LAI groups that initiated an LAI reactively or proactively; proportion of patients with successful LAI implementation (≥90 days of continuous treatment with ≤7-day gaps); time to successful LAI implementation; and schizophrenia-related HCRU and costs within 12 months after first LAI administration.

### Statistical analysis

All outcomes were examined descriptively. Mann–Whitney U and Chi-square/Fisher’s exact tests were used to test statistical significance for continuous and categorical values, respectively. All outcomes and costs were annualized and reported as per patient per year, and costs were standardized to 2020 $USD.

## Results

**Fig. 1:**
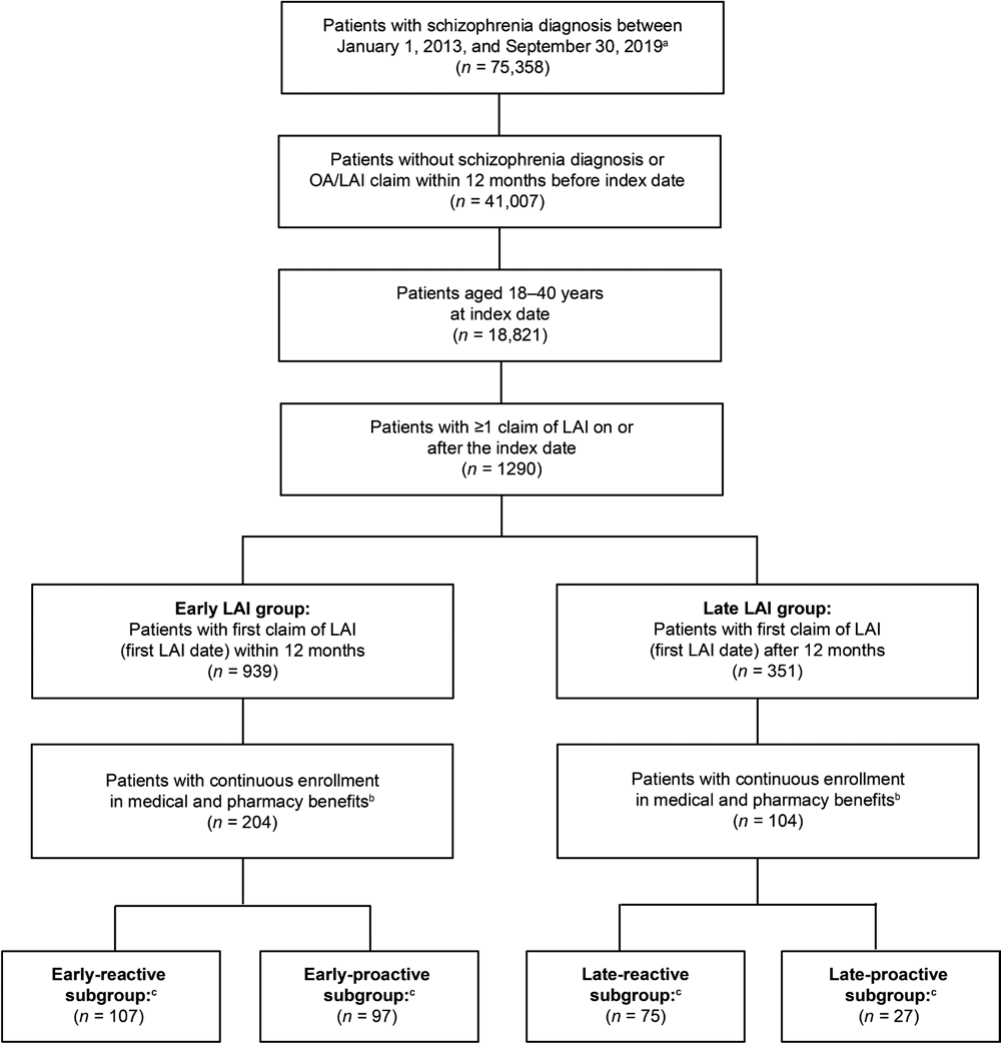
Selection of study population. ED, emergency department; LAI, long-acting injectable antipsychotic agent; OA, oral antipsychotic agent. ^a^The first diagnosis claim is the index date. ^b^Enrolled 12 months before the index date and in the 12 months following initiation of first LAI (≤30-day gaps allowed). ^c^Patients with (reactive) or without (proactive) ≥1 schizophrenia-related hospitalization or ED visit before LAI initiation.

Of the 75 358 individuals with a schizophrenia diagnosis between 1 January 2013 and 30 September 2019, 1821 were 18–40 years old with no schizophrenia diagnosis and no claims filed for an OA or LAI during the 12 months preceding the index date (i.e. newly diagnosed schizophrenia) (Fig. 1). Among these, 1290 had ≥1 LAI claim, and 306 met continuous benefit enrollment criteria (Fig. 1). Of these, 204 and 102 met the criteria for the early LAI and late LAI groups, respectively (Fig. 1). Most were men (early, 76.9%; late, 82.4%) and mean (SD) age was 23.3 (4.6) and 23.9 (5.9) years, respectively (Table 1). The average Charlson Comorbidity Index (higher score predicts an increased risk of death from comorbid disease) (Charlson *et al.*, 1987, 2008) was 0.10 (SD, 0.3) for the entire cohort (Table 1). The most frequently administered first LAIs were paliperidone (early, 56.4%; late, 57.8%), aripiprazole (22.6%; 32.4%), risperidone (19.1%; 7.8%), and olanzapine (2.0%; 2.0%) (Table 1).

**Table 1:** Patient, demographics

Characteristic	LAIs Overall	Early LAI^a^	Late LAI^b^	*P* value
	*N* = 306	*n* = 204	*n* = 102	
Male *n* (%)	241 (78.8)	157 (76.9)	84 (82.4)	0.2770
Age, years, mean (SD)	23.5 (5.0)	23.3 (4.6)	23.9 (5.9)	0.5428
Age group, years, *n* (%)				
18–23	208 (68.0)	140 (68.6)	68 (66.7)	0.3502
24–29	60 (19.6)	43 (21.1)	17 (16.7)	
30–35	22 (7.2)	13 (6.4)	9 (8.8)	
36–40	16 (5.2)	8 (3.9)	8 (7.8)	
CCI score,^c^ mean (SD)	0.10 (0.3)	0.10 (0.3)	0.12 (0.3)	0.4349
First LAI used, *n* (%)				
Paliperidone	174 (56.9)	115 (56.4)	59 (57.8)	0.0303
Aripiprazole	79 (25.8)	46 (22.6)	33 (32.4)	
Risperidone	47 (15.4)	39 (19.1)	8 (7.8)	
Olanzapine	6 (2.0)	4 (2.0)	2 (2.0)	
Insurance type, *n* (%)				
PPO	158 (51.6)	113 (55.4)	45 (44.1)	0.3747
CDHP	40 (13.1)	28 (13.7)	12 (11.8)	
HMO	30 (9.8)	16 (7.8)	14 (13.7)	
Noncapitated PPO	23 (7.5)	14 (6.9)	9 (8.8)	
Basic/Major Medical	22 (7.2)	15 (7.4)	7 (6.9)	
Comprehensive	22 (7.2)	12 (5.9)	10 (9.8)	
EPO	2 (0.7)	1 (0.5)	1 (1.0)	
POS	1 (0.3)	1 (0.5)	0	
Other	8 (2.6)	4 (2.0)	4 (3.9)	
Region, *n* (%)				
South	113 (36.9)	79 (38.7)	34 (33.3)	0.3955
North Central	92 (30.1)	62 (30.4)	30 (29.4)	
Northeast	50 (16.3)	30 (14.7)	20 (19.6)	
West	47 (15.4)	29 (14.2)	18 (17.7)	
Unknown	4 (1.3)	4 (2.0)	0	

Significant *P* values are bolded.

CCI, Charlson Comorbidity Index; CDHP, consumer-directed health plan; EPO, exclusive provider organization; HMO, health maintenance organization; LAI, long-acting injectable antipsychotic agent; POS, point of service; PPO, preferred provider organization.

a First LAI claim ≤1 year after index date.

b First LAI claim >1 year after index date.

c For preindex period (12 months before index date).

Patients in the early and late LAI groups were then divided based on whether LAIs were initiated reactively (i.e. after ≥1 schizophrenia-related hospitalization or ED visit) or proactively (i.e. not after schizophrenia-related hospitalization or ED visit). In the early and late LAI groups, 107 patients and 75 patients started LAIs reactively, and 97 patients in the early LAI group and 27 patients in the late LAI group started LAIs proactively.

Proportions of patients with successful LAI implementation, defined as at least 90 days of continuous use with an allowance of 7 days or less for refills, were 53.9 and 48.0% in the early and late groups, respectively. Median [interquartile range (IQR)] times to successful LAI implementation were 177 (153) and 184 (237) days. Higher proportion of patients in the late-reactive subgroup had ≥1 and ≥2 schizophrenia-related hospitalizations or ED visits before LAI initiation versus those in the early-reactive subgroup; conversely, lower proportions in the late-proactive subgroup compared with the early-proactive subgroup had ≥1 and ≥2 schizophrenia-related hospitalizations or ED visits before LAI initiation (Table 2). Implementation of LAI therapy was successful in approximately half of the schizophrenia cohort (52.0%; *n* = 159); median (IQR) time to successful implementation was 180 (171) days from diagnosis. Among patients who initiated LAIs reactively, it took a median (IQR) of 45.0 (96.0) days for the early group compared with 525.0 (414.0) days for the late group (Table 2). Patients who initiated an LAI late and after at least one schizophrenia-related hospitalization or ED visit (i.e. late-reactive) took approximately 10 times longer to initiate LAI therapy after the index date [median (IQR), 608.0 (502.0) days] than those who initiated an LAI early [59.0 (148.0) days] (Table 2).

**Table 2:** LAI initiation timeline versus schizophrenia-related hospitalization or ED visit

Schizophrenia-related hospitalization or ED visit before LAI initiation	LAIs overall	Early LAI^a^		Late LAI^b^	
		*n* = 204		*n* = 102	
	*N* = 306	Reactive	Proactive	Reactive	Proactive
Patient with ≥1 schizophrenia-related hospitalization or ED visit before LAI initiation^c^, *n* (%)	182 (59.5)	107 (52.5)	97 (47.6)	75 (73.5)	27 (26.5)
Patients with ≥2 schizophrenia-related hospitalization or ED visit before LAI initiation, *n* (%)	79 (25.8)	30 (14.7)	174 (85.3)	49 (48.0)	53 (52.0)
Days between index date and 1st schizophrenia-related hospitalization or ED visit					
Mean (SD)	63.6 (173.2)	18.0 (61.3)	–	128.6 (246.3)	–
Median (IQR)	0 (1)	0	–	0 (131)	–
Days between 1st schizophrenia-related hospitalization or ED visit and LAI initiation^d^					
Mean (SD)	300.3 (357.7)	91.8 (94.8)	–	597.7 (384.1)	–
Median (IQR)	134.0 (424.0)	45.0 (96.0)	–	525.0 (414.0)	–
Days between index date and LAI initiation					
Mean (SD)	307.9 (362.4)	109.8 (104.5)	86.8 (98.4)	726.3 (340.8)	724.7 (317.2)
Median (IQR)	136.0 (432.0)	59.0 (148.0)	46.0 (100.0)	608.0 (502.0)	615.0 (380.0)

–, not applicable; ED, emergency department; IQR, interquartile range; LAI, long-acting injectable antipsychotic agent.

a First LAI claim ≤1 year after index date.

b First LAI claim >1 year after index date.

c Between index and initiation date, including the index date, but not including LAI initiation date.

d Average days to LAI initiation was calculated based on patients who have schizophrenia-related hospitalization or ED visit.

Although overall mean (SD) costs were found to be numerically lower for the early [$7088.91 ($13 753.61)] versus late [$9389.94 ($20 792.25)] LAI groups, differences were not statistically significant (Supplementary Table 1, Supplementary Fig. 2, Supplemental Digital Content 1, http://links.lww.com/ICP/A112). Mean (SD) schizophrenia-related HCRU and costs per patient were lower in the early LAI group than in the late group for ED visits [early, 0.1 (0.5) vs. late, 0.2 (0.7); $140.50 ($661.25) vs. $173.21 ($610.08)] and other outpatient visits [3.2 (9.1) vs. 4.0 (12.9); $1140.18 ($3982.48) vs. $2373.01 ($8817.03)], and greater for office visits [4.3 (8.9) vs. 3.7 (6.1); $1219.86 ($4965.38) vs. $1061.05 ($3972.94)] (Supplementary Table 1, Supplementary Fig. 2, Supplemental Digital Content 1, http://links.lww.com/ICP/A112). Although antipsychotic prescription utilization was numerically lower in the early group than in the late group [4.7 (7.2) vs. 5.4 (7.5)], the differences were not statistically significant. Antipsychotic prescription costs were comparable among the early and late groups [$1375.34 ($3774.01) vs. $1365.61 ($2538.26)] (Supplementary Table 1, Supplementary Fig. 2, Supplemental Digital Content 1, http://links.lww.com/ICP/A112).

**Fig. 2:**
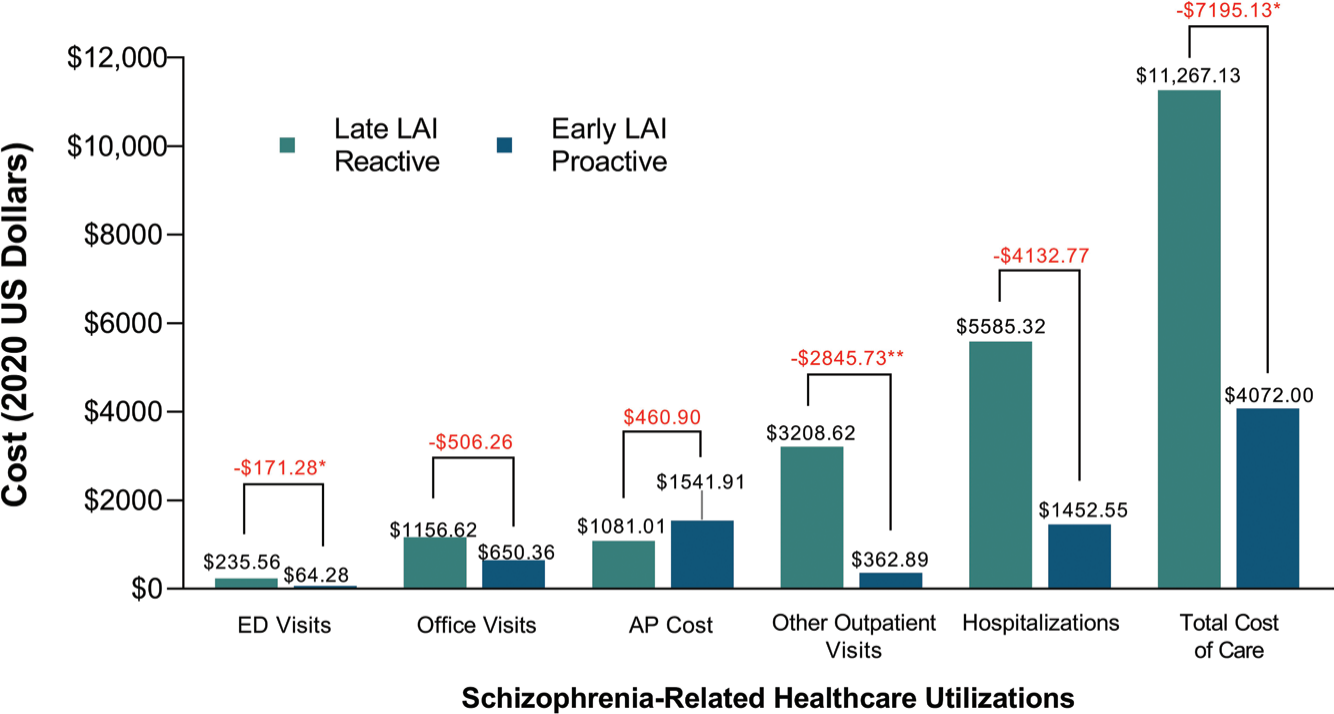
Late-reactive versus early-proactive schizophrenia-related healthcare costs per patient. Early LAI, first LAI claim ≤1 year after index date. Late LAI, first LAI claim >1 year after index date. Proactive, no schizophrenia-related hospitalization or ED visit before LAI initiation. Reactive, schizophrenia-related hospitalization or ED visit prior to LAI initiation. *P < 0.05 **P < 0.00001. AP, antipsychotic agent; ED, emergency department; LAI, long-acting injectable antipsychotic agent.

A lower proportion of the early group (*n* = 107; 52.5%) initiated an LAI reactively compared with the late group (*n* = 75; 73.5%; *P* = 0.0004) (Table 2). In the early and late groups, proportions of patients with at least one schizophrenia-related hospitalization after-LAI initiation were higher for patients who initiated an LAI reactively than for those who initiated it proactively (early, 20.6% vs. 10.3%, *P* = 0.0444; late, 20.0% vs. 7.4%, *P* = 0.2266) (Table 3). Proportions of patients with other outpatient visits were significantly different between reactive and proactive subgroups in early (46.7% vs. 20.6%; *P* = 0.0001) and late groups (50.7% vs. 25.9%; *P* = 0.0264) (Table 3). In early and late LAI groups, overall healthcare utilizations were lower in the proactive subgroups than those in the reactive subgroups. This translated into average total costs of schizophrenia-related healthcare differences of $5751.87 (*P* = 0.0086) and $7091.60 (*P* = 0.4851) between the reactive and the proactive subgroups for early and late groups, respectively (Table 3, Supplementary Fig. 3, Supplemental Digital Content 1, http://links.lww.com/ICP/A112). Comparing the worst-case scenario (late-reactive) to the best scenario (early-proactive), the average cost difference was $7195.13 (*P* = 0.0233) (Table 3, Supplementary Fig. 3, Supplemental Digital Content 1, http://links.lww.com/ICP/A112). For this comparison between late-reactive versus early-proactive, cost difference was greatest for ED visits ($171.28; *P* < 0.0154) and other outpatient visits ($2845.73; *P* < 0.00001) (Table 3 and Fig. 2). The reactive subgroups had lower annual antipsychotic medication costs than the proactive subgroups, with the difference in the early group being lower than in the late group ($317.58 vs. $1075.16, respectively). Differences in antipsychotic medication use and cost between reactive and proactive subgroups in the late LAI group were significant where the proactive subgroup had a greater proportion of patients with ≥1 prescription (*P* = 0.0200) and higher costs (*P* = 0.0201).

**Table 3:** Reactive versus proactive schizophrenia-related healthcare resource utilization and costs during the 12 months after LAI initiation

Schizophrenia-related healthcare resource utilization or cost after LAI initiation	Early LAI^a^, *n* = 204			Late LAI^b^, *n* = 102			Late LAI reactive vs. early LAI proactive
	Reactive	Proactive	*P* value	Reactive	Proactive	*P* value	*P* value
	*n* = 107	*n* = 97		*n* = 75	*n* = 27		
Schizophrenia-related hospitalization							
Hospitalization per patient, mean (SD)	0.3 (0.7)	0.1 (0.4)	0.0303	0.3 (0.7)	0.1 (0.3)	0.1249	0.0654
Patients with ≥1 hospitalization, *n* (%)	22 (20.6)	10 (10.3)	0.0444	15 (20.0)	2 (7.4)	0.2266	0.0737
Hospitalization costs per patient, $2020 USD, mean (SD)	4809.00 (13 029.54)	1452.55 (5586.33)	0.0314	5585.32 (19 518.02)	1171.69 (5385.94)	0.1347	0.0631
Readmissions per patient, mean (SD)	0.04 (0.2)	0	0.0557	0.1 (0.3)	0	0.2275	0.0221
Patients with ≥1 readmission,^c^ *n* (%)	4 (3.7)	0	0.1231	4 (5.3)	0	0.5710	0.0345
Schizophrenia-related ED visits							
ER visits per patient, mean (SD)	0.2 (0.7)	0.1 (0.4)	0.2140	0.3 (0.9)	0	0.0370	0.0152
Patients with ≥1 ER visit, *n* (%)	9 (8.4)	4 (4.1)	0.2106	11 (14.7)	0	0.0345	0.0151
ED costs per patient, $2020 USD, mean (SD)	209.58 (846.87)	64.28 (348.61)	0.2029	235.56 (702.22)	0	0.0371	0.0154
Schizophrenia-related office visits							
Office visits per person, mean (SD)	5.1 (10.2)	3.3 (7.1)	0.6983	3.6 (5.5)	3.8 (7.7)	0.5854	0.3324
Patients with ≥1 office visit, *n* (%)	49 (45.8)	47 (48.5)	0.7039	40 (53.3)	12 (44.4)	0.4282	0.5256
Office visit costs per person, $2020 USD, mean (SD)	1736.13 (6574.46)	650.36 (1945.33)	0.6535	1156.62 (4469.99)	795.59 (2085.11)	0.5474	0.3228
Schizophrenia-related other outpatient visits							
Other outpatient visits per patient, mean (SD)	4.9 (11.5)	1.4 (4.7)	0.0001	5.4 (14.8)	0.3 (0.7)	0.0062	0.0001
Patients with ≥1 other outpatient visit, *n* (%)	50 (46.7)	20 (20.6)	0.0001	38 (50.7)	7 (25.9)	0.0264	<0.0001
Other outpatient visit costs per patient, $2020 USD, mean (SD)	1844.83 (5231.85)	362.89 (1466.34)	<0.0001	3208.62 (10 169.80)	52.08 (137.94)	0.0063	<0.0001
Antipsychotic agents							
AP per patient, mean (SD)	4.5 (7.4)	4.9 (7.1)	0.2308	4.9 (7.3)	6.8 (7.8)	0.0482	0.6824
Patients with ≥1 prescription, *n* (%)	67 (62.6)	67 (69.1)	0.3321	49 (65.3)	24 (88.9)	0.0200	0.6038
AP costs per patient, $2020 USD, mean (SD)	1224.33 (3154.62)	1541.91 (4367.67)	0.3342	1081.01 (2287.54)	2156.17 (3041.64)	0.0201	0.4993
Total cost of care for schizophrenia							
Total schizophrenia-related healthcare costs per patient, $2020 USD, mean (SD)	9823.87 (16 771.45)	4072.00 (8478.38)	0.0086	11 267.13 (23 741.67)	4175.53 (6058.40)	0.4581	0.0233

Significant *P* values are bolded.

AP, antipsychotic agent; ED, emergency department; LAI, long-acting injectable antipsychotic agent.

a First LAI claim ≤1 year after index date.

b First LAI claim >1 year after index date.

c Within ≤30 days after discharge.

Main drivers for cost savings between reactive and proactive subgroups were other outpatient visits and hospitalizations costs: the reactive subgroup had $1481.94 (*P* < 0.00001) and $3156.54 (*P* = 0.0063) higher annual other outpatient costs in the early and late groups, respectively (Table 3). Comparing late-reactive to early-proactive subgroups, the average cost difference was $2845.73 (*P* < 0.00001) (Table 3 and Fig. 2). The largest significant differences in other outpatient and hospitalizations costs between the reactive and proactive subgroups were seen in the late LAI group where the reactive group had $3156.54 (*P* = 0.0063) and $4413.63 (*P* = 0.1347) higher costs for outpatient visits and hospitalization, respectively (Table 3). The early LAI group had significant differences in hospitalization costs where the reactive subgroup had $3356.45 (*P* = 0.0314) higher annual hospitalizations costs than the proactive subgroup (Table 3).

## Discussion

Using a US-based commercial claims database, this study evaluated treatment patterns, HCRU, and costs among patients with newly diagnosed schizophrenia who began treatment with LAIs early (≤1 year) versus late (>1 year) following diagnosis. These data reveal that early LAI initiation can lead to more patients with successful implementation (≥90 days of continuous adherence) of LAI therapy. This aligns with several recent studies demonstrating that LAIs effectively improved daily adherence, and even more so when the LAI was initiated at illness onset or early in the diagnosis (Subotnik *et al.*, 2015; Stevens *et al.*, 2016; Greene *et al.*, 2018).

The proportion of healthcare resource use, such as hospital admissions, readmissions, ED visits, and other outpatient visits, was lower in the early LAI group than in the late LAI group. In addition, the early LAI group had lower schizophrenia-related costs per person than the late LAI group. This cost difference was primarily related to differences in hospitalization and other outpatient visit costs. These results align with a previous study that found a reduction in healthcare costs in a US-based commercially insured schizophrenia population after the initial diagnosis was largely driven by a reduction in hospitalizations and ED visits (Fitch *et al.*, 2014). Furthermore, LAI use in early-phase schizophrenia significantly reduced the incidence rate of hospitalization after their implementation, which is an important economic outcome (Kane *et al.*, 2020). Our assessment of total cost of care for schizophrenia showed an unadjusted average of $2301.03 in cost savings among the early group compared with the late LAI group. However, neither differences in utilizations nor costs between early LAI and late LAI groups were found to be statistically significant.

The results from this study suggest that patients with schizophrenia are not offered LAI therapy in a timely manner as demonstrated by the substantially lengthier time (10 times longer) for patients in the late LAI group to initiate an LAI after at least 1 schizophrenia-related high-risk event (median, 608 days); patients in the early LAI group that initiated an LAI reactively did so considerably more quickly (median, 59 days). There are several reasons why a clinician may be reluctant to initiate an LAI in a patient with newly diagnosed schizophrenia, including efficacy and tolerability concerns, reluctance to deviate from earlier treatment guidelines, expert opinions that advised OAs as first-line treatment, and limited availability of atypical LAIs on the market (Kirschner *et al.*, 2013). In addition, a recent real-world survey found that clinicians tended to underestimate disease severity and overestimate medication adherence, leading to suboptimal treatment and poor patient outcomes (Keenan *et al.*, 2022). However, in a cluster-randomized LAI clinical trial, in which participating investigators were required to undergo training about the study protocol and procedures, as well as training on how to discuss frequently asked questions with patients being offered LAI therapy, 91.0% of patients with first-episode or early-phase schizophrenia were willing to try LAI therapy when the option to do so was presented in a supportive manner by trained staff (Kane *et al.*, 2019).

For early and late LAI groups, HCRU was lower among the proactive subgroup than the reactive subgroup. Antipsychotic medication costs did not differ greatly between early and late groups, which can counteract arguments against LAI use because of high medication costs. Although medication costs were lower in the reactive subgroups than in the proactive subgroups, the early-proactive subgroup had the lowest cost difference. Proactive LAI utilization, regardless of early or late initiation, was associated with lower HCRU and costs than reactive use; this was largely driven by ED visits and other outpatient visits. The largest significant differences in other outpatient visits and hospitalization costs between reactive and proactive subgroups were seen in the late LAI group, suggesting that proactive use, even if initiated late, would still provide cost savings. Overall, the early-proactive LAI subgroup had the lowest annual average schizophrenia-related costs, $4072.00 (8478.38), whereas the late-reactive LAI subgroup had the highest annual schizophrenia-related costs, $11 267.13 (23 741.67).

These data show that LAI implementation is generally associated with lower HCRU and costs among patients who received an LAI within 1 year of a schizophrenia diagnosis versus with those who initiated treatment with an LAI more than 1 year after diagnosis. Treatment guidelines for schizophrenia management recommend that LAIs be considered for initial therapy instead of reserving them strictly for patients who have demonstrated nonadherence or have had multiple relapses (Florida Medicaid Drug Therapy Management Program, 2020; Keepers *et al.*, 2020). LAIs are a highly effective treatment option that offer a greater likelihood of stability for many patients with schizophrenia (Kishimoto *et al.*, 2013, 2021). In real-world studies, LAIs have proved superior to OAs, lowering the risk of relapse, hospitalization, and all-cause discontinuation risk, despite greater illness severity in patients who initiate LAIs versus OAs (Kishimoto *et al.*, 2013, 2021). Initiation of LAIs should be considered based on a combination of patient preference, patient education, and outcomes evidence (Florida Medicaid Drug Therapy Management Program, 2020; Keepers *et al.*, 2020). These data support the early use of LAIs in newly diagnosed schizophrenia and demonstrate early use of LAIs corresponding to lower HCRU and schizophrenia-related healthcare costs.

There were several limitations in this study. Although young adults are more likely to have commercial insurance than Medicaid or Medicare, which are often utilized by older patients, claims data presented here are more reflective of younger patients with earlier-stage disease than earlier studies based on public insurance claims (Cohen *et al.*, 2020). However, commercial claims data are still not entirely representative of all patients with early-phase schizophrenia. The 1-year cutoff timeframe to distinguish between early and late LAI initiation may be arbitrary since early use of an LAI has been variably defined as 1–5 years after initial schizophrenia diagnosis (Sliwa *et al.*, 2012; Munday *et al.*, 2019). However, the 1-year cutoff used in this study was based on a prior study evaluating LAI utilization among patients with schizophrenia that found that the median time between diagnosis and LAI initiation was approximately 290 days (Kane *et al.*, 2021). In addition, the 1-year cutoff point was used in a previous study also looking at cost outcomes among patients with schizophrenia (Munday *et al.*, 2019). The use of an LAI was regarded as reactive if initiated after at least one schizophrenia-related hospitalization or ED visit. However, it was possible that an LAI was used ‘reactively’ if a patient required a change in treatment without the need for hospitalization or an ED visit. The results of this analysis are descriptive in nature and are not adjusted for covariates such as patient demographics and schizophrenia-related factors. The inability to randomize the various conditions in the study (i.e. early vs. late initiation, reactive vs. proactive), prevents making causal interpretations. However, these results align with those of the PRELAPSE study, in which individuals were cluster-randomized by clinic to LAI prescriber training versus usual care, and it was found that the use of LAIs early in the course of illness was associated with greater clinical stability (Kane *et al.*, 2019).

Overall, the study provides cost-based evidence in favor of early use of LAIs for patients with newly diagnosed schizophrenia with potential additive benefits if patients start LAIs proactively. Early intervention with LAI treatment could be an effective method for reducing schizophrenia-related hospital admission or readmission, as well as ED admission and other outpatient visits, ultimately resulting in the potential for substantial cost-savings, although these associations need to be tested in rigorous, randomized prospective trials.

## Acknowledgements

Medical writing support was provided by Amanda Cox, PhD, and Jennifer C. Jaworski, MS, BCMAS, and editorial support by Kelsey Hogan, MS, of Ashfield MedComms, an Inizio company, and was funded by Teva Branded Pharmaceutical Products R&D, Inc.

### Conflicts of interest

J.M.H. has been a consultant for or received honoraria from Alkermes, EnVivo Pharmaceuticals (Forum), Forest (Allergan), Genentech, Intra-Cellular Therapies, Janssen, Johnson & Johnson, Karuna Therapeutics, LB Pharmaceuticals, Lilly, Lundbeck, Lyndra Therapeutics, Merck, Neurocrine Biosciences, Otsuka, Pierre Fabre, Reviva Pharmaceuticals, Roche, Saladax Biomedical, Sunovion, Takeda, and Teva Pharmaceuticals; has received grant support from Janssen, Lundbeck, and Otsuka; and is a shareholder of LB Pharmaceuticals and Vanguard Research Group. A.C. was a post-doctoral fellow at Teva Branded Pharmaceutical Products R&D, Inc. at the time of this research. S.L. is an employee of Teva Pharmaceuticals. M.A.M, M.T., Y.W., and M.S. are employees and stockholders of Teva Pharmaceuticals. J.M.R. has been a consultant for and has received support for attending meetings/travel from Teva Pharmaceuticals; has received honoraria from Lundbeck; has received grants from Alkermes and the National Institute of Mental Health (NIMH); has received royalties/licensing fees from UpToDate; and owns stock/stock options in Doximity.

## Supplementary Material


