# Supplementary material for: Early versus late administration of long-acting injectable antipsychotic agents among patients with newly diagnosed schizophrenia: an analysis of a commercial claims database
Source: Int Clin Psychopharmacol. 2023 Feb 8;38(4):240–8. doi: 10.1097/YIC.0000000000000452 (PMC10234320; doi:10.1097/YIC.0000000000000452)
Supplement: Supplementary file 1 [file icp-38-240-s001.pdf]

## SUPPLEMENTARY MATERIALS

### Supplementary Table 1 Schizophrenia-related healthcare resource utilization and costs 12 months post-LAI initiation

| | LAIs Overall *N*=306 | Early LAI[a] *n*=204 | Late LAI[b] *n*=102 | Early LAI vs Late LAI *P* Value |
|---|---|---|---|---|
| **Schizophrenia-related hospitalization** | | | | |
| Hospitalization per patient, mean (SD) | 0.2 (0.6) | 0.2 (0.6) | 0.2 (0.6) | 0.8863 |
| Patients with ≥1 hospitalization, *n* (%) | 49 (16.0) | 32 (15.7) | 17 (16.7) | 0.8255 |
| Hospitalization costs per patient, $2020 USD, mean (SD) | 3614.36 (12,930.41) | 3213.04 (10,306.76) | 4417.01 (17,041.44) | 0.8292 |
| Readmissions per patient, mean (SD) | 0.03 (0.2) | 0.02 (0.1) | 0.05 (0.3) | 0.3082 |
| Patients with ≥1 readmission,[c] *n* (%) | 8 (2.6) | 4 (2.0) | 4 (3.9) | 0.4479 |
| **Schizophrenia-related ED visits** | | | | |
| ED visits per patient, mean (SD) | 0.2 (0.6) | 0.1 (0.5) | 0.2 (0.7) | 0.1738 |
| Patients with ≥1 ED visit, *n* (%) | 24 (7.8) | 13 (6.4) | 11 (10.8) | 0.1760 |
| ED costs per patient, $2020 USD, mean (SD) | 151.40 (643.83) | 140.50 (661.25) | 173.21 (610.08) | 0.1898 |
| **Schizophrenia-related office visits** | | | | |
| Office visits per person, mean (SD) | 4.1 (8.1) | 4.3 (8.9) | 3.7 (6.1) | 0.5385 |
| Patients with ≥1 office visit, *n* (%) | 148 (48.4) | 96 (47.1) | 52 (51.0) | 0.5176 |
| Office visit costs per person, $2020 USD, mean (SD) | 1166.92 (4652.13) | 1219.86 (4965.38) | 1061.05 (3972.94) | 0.5598 |
| **Schizophrenia related other outpatient visits** | | | | |
| Other outpatient visits per patient, mean (SD) | 3.5 (10.5) | 3.2 (9.1) | 4.0 (12.9) | 0.2428 |
| Patients with ≥1 other outpatient visit, *n* (%) | 115 (37.6) | 70 (34.3) | 45 (44.1) | 0.0951 |

| | | | | |
|---|---|---|---|---|
| Other outpatient visit costs per patient, $2020 USD, mean (SD) | 1551.14 (6052.97) | 1140.18 (3982.48) | 2373.07 (8817.03) | 0.1599 |
| **Antipsychotic agents** | | | | |
| AP per patient, mean (SD) | 4.9 (7.3) | 4.7 (7.2) | 5.4 (7.5) | 0.2424 |
| Patients with ≥1 prescription, *n* (%) | 207 (67.7) | 134 (65.7) | 73 (71.6) | 0.2998 |
| AP costs per patient, $2020 USD, mean (SD) | 1372.10 (3407.84) | 1375.34 (3774.01) | 1365.61 (2538.26) | 0.4314 |
| **Total cost of care for schizophrenia** | | | | |
| Total schizophrenia–related healthcare costs per patient, $2020 USD, mean (SD) | 7855.92 (16,439.05) | 7088.91 (13,753.61) | 9389.94 (20,792.25) | 0.3771 |

AP, antipsychotic agent; ED, emergency department; LAI, long-acting injectable antipsychotic agent; SD, standard deviation.
[a]First LAI claim ≤1 year after index date. [b]First LAI claim >1 year after index date. [c]Within ≤30 days after discharge.

**Supplementary Fig. 1 Study Design**

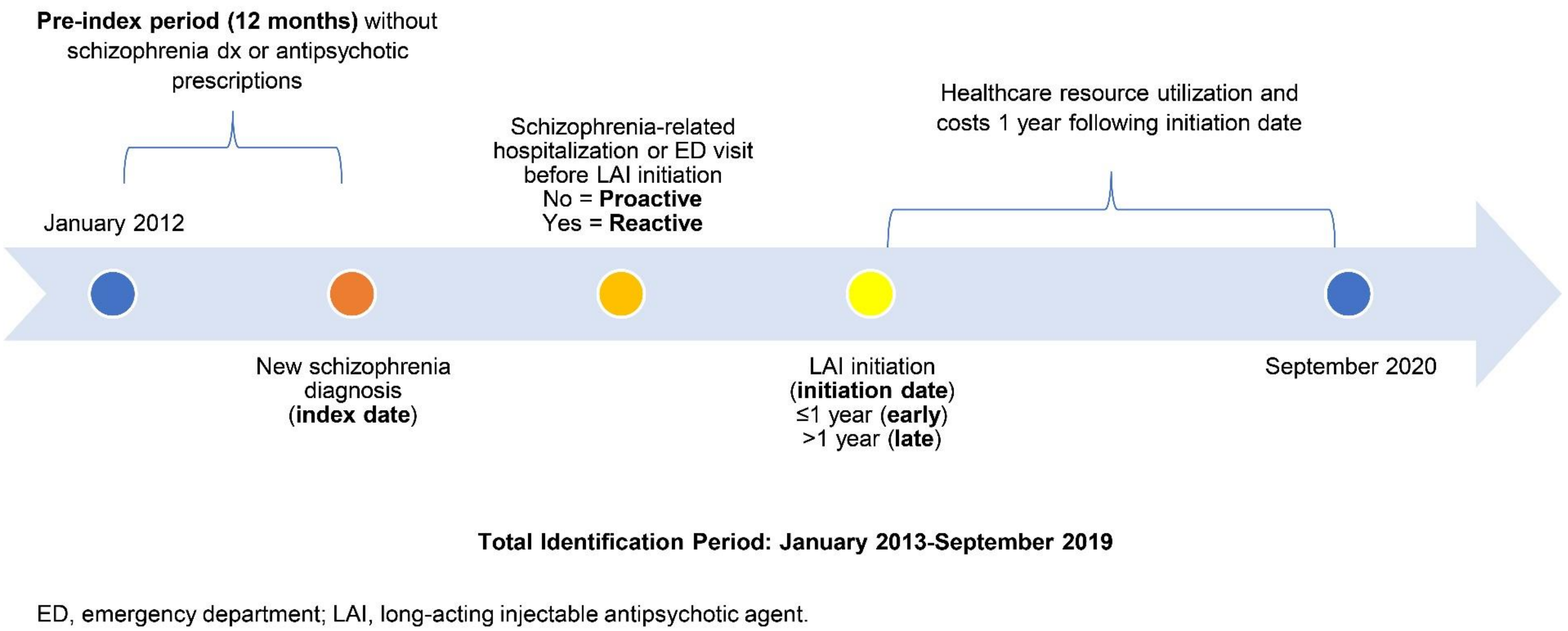


ED, emergency department; LAI, long-acting injectable antipsychotic agent.

**Supplementary Fig. 2 Schizophrenia-Related Healthcare Costs Per Patient for Early Vs Late LAI Initiation**

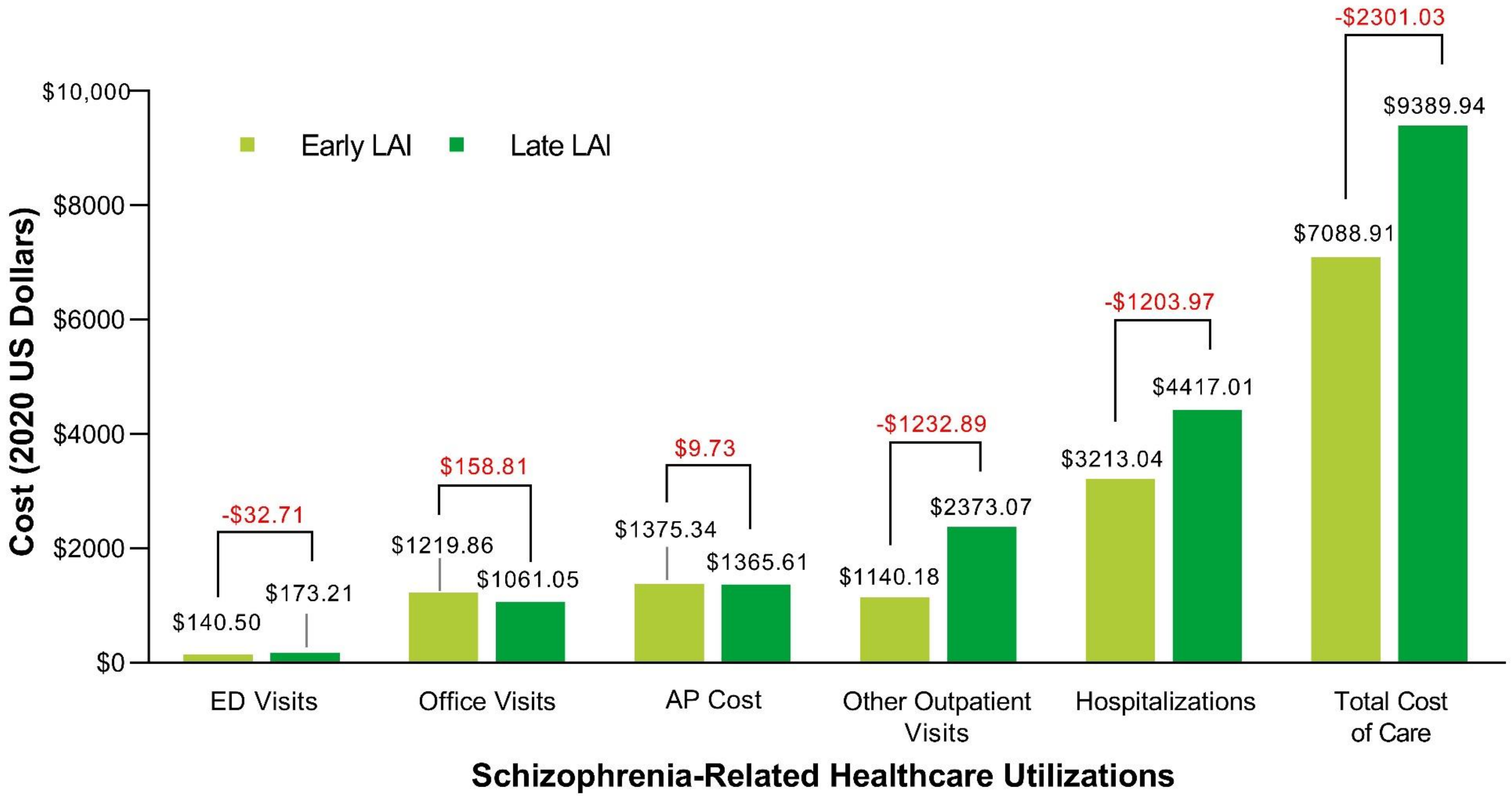


AP, antipsychotic agent; ED, emergency department; LAI, long-acting injectable antipsychotic agent.
Early LAI, first LAI claim ≤1 year after index date. Late LAI, first LAI claim >1 year after index date.

**Supplementary Fig. 3 Total Schizophrenia-Related Healthcare Costs Per Patient During First 12 Months After LAI Initiation**

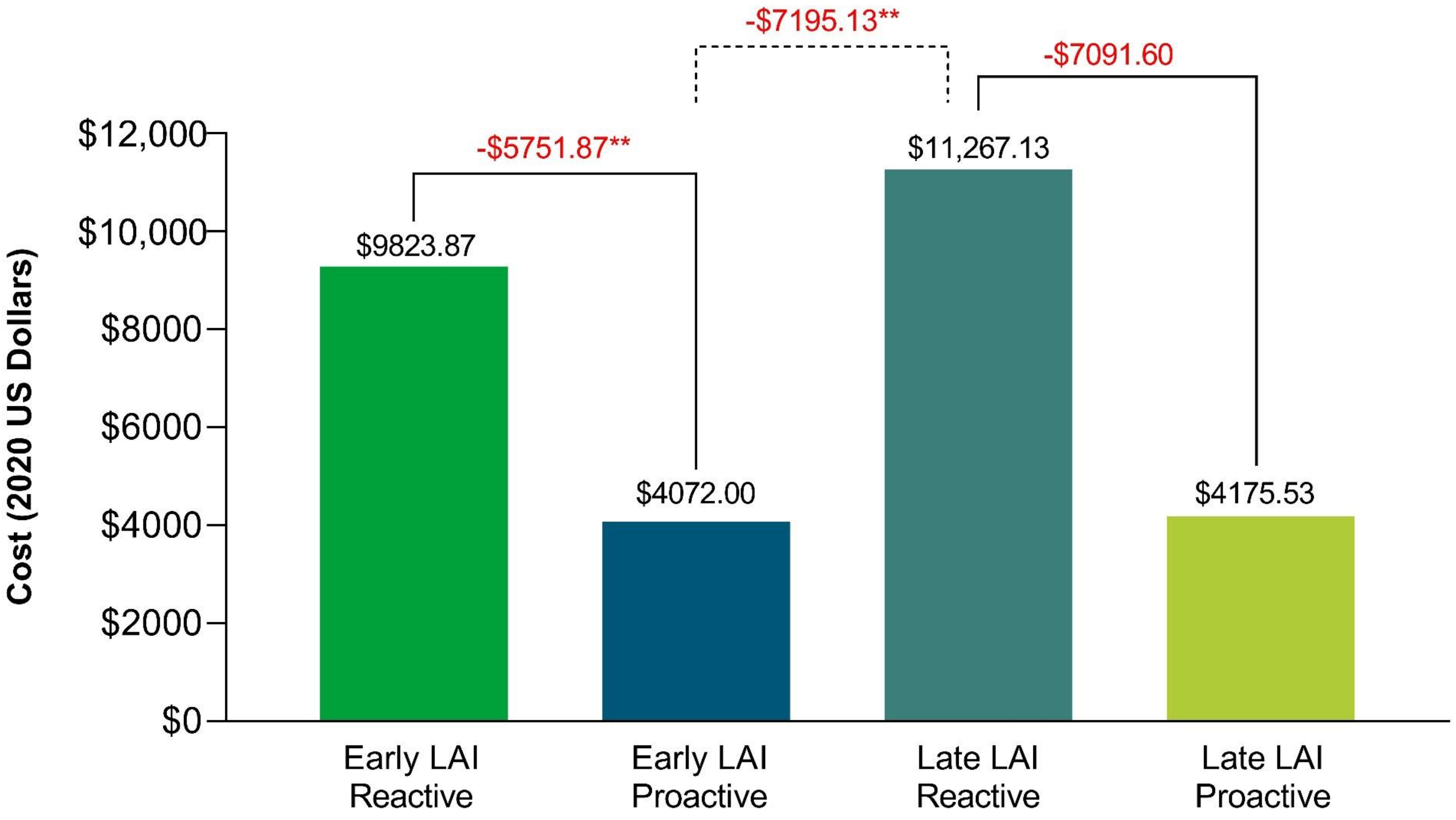


LAI, long-acting injectable antipsychotic agent.
Early LAI, first LAI claim ≤1 year after index date. Late LAI, first LAI claim >1 year after index date. Proactive, no schizophrenia-related hospitalization or ED visit before LAI initiation. Reactive, schizophrenia-related Hospitalization or ED visit prior to LAI initiation.
**$P$<0.05
